# Modeling of Severity Classification Algorithm Using Abdominal Aortic Aneurysm Computed Tomography Image Segmentation Based on U-Net with Improved Noise Reduction Performance

**DOI:** 10.3390/s25216509

**Published:** 2025-10-22

**Authors:** Sewon Lim, Hajin Kim, Kang-Hyeon Seo, Youngjin Lee

**Affiliations:** 1 Department of Health Science, General Graduate School of Gachon University, 191, Hambakmoe-ro, Yeonsu-gu, Incheon 21936, Republic of Korea; tpdnjs728@gachon.ac.kr (S.L.); happida3@gachon.ac.kr (H.K.); 2 Department of Radiology, Hallym Hospital, 722, Jangjero, Gyeyang-gu, Incheon 21079, Republic of Korea; secrefer@naver.com; 3 Department of Radiological Science, Gachon University, 191, Hambakmoe-ro, Yeonsu-gu, Incheon 21936, Republic of Korea

**Keywords:** abdominal aortic aneurysm, U-Net, median modified Wiener filter, noise reduction algorithm, severity classification

## Abstract

**Highlights:**

**What are the main findings?**

- The application of the median-modified Wiener filter significantly improved the U-Net-based segmentation performance on abdominal aortic aneurysm CT images with added Poisson–Gaussian noise.
- Segmentation quality directly influenced automated severity classification performance. When MMWF was used as a preprocessing step, the Hough circle-based classification achieved 100% sensitivity, precision, and accuracy.

**What is the implication of the main finding?**

- Integrating classical filtering methods into deep learning-based segmentation pipelines can enhance robustness against noise without modifying network architecture.
- Accurate segmentation via preprocessing contributes to more reliable automated severity classification of AAAs, supporting improved clinical decision-making in noisy imaging conditions.

**Abstract:**

Accurate segmentation of abdominal aortic aneurysm (AAA) from computed tomography (CT) images is critical for early diagnosis and treatment planning of vascular diseases. However, noise in CT images obscures vessel boundaries, reducing segmentation accuracy. U-Net is widely used for medical image segmentation, where noise removal is critical. This study applied various denoising filters for U-Net segmentation and classified the severity of segmented AAA images to evaluate accuracy. Poisson–Gaussian noise was added to AAA CT images, and then average, median, Wiener, and median-modified Wiener filters (MMWF) were applied. U-Net-based segmentation was performed, and the segmentation accuracy of the output images obtained per filter was quantitatively assessed. Furthermore, the Hough circle algorithm was applied to the segmented images for diameter measurement, enabling severity classification and evaluation of classification accuracy. MMWF application improved the Matthews correlation coefficient, Dice score, Jaccard coefficient, and mean surface distance by 31.09%, 34.25%, 53.99%, and 3.70%, respectively, compared with images with added noise. Moreover, classification based on the output images obtained after MMWF application demonstrated the highest accuracy, with sensitivity, precision, and accuracy reaching 100%. Thus, U-Net-based segmentation yields more accurate results when images are processed with the MMWF and analyzed using the Hough circle algorithm.

## 1. Introduction

An abdominal aortic aneurysm (AAA) is characterized by localized enlargement of the aorta between the renal and iliac arteries. AAA rupture accounts for 1.3% of deaths in men aged > 65 [1,2]. The development of AAA involves structural changes in the aortic wall, including thinning of the intima due to extracellular matrix degradation within the arterial intima and loss of vascular smooth muscle cells [3]. AAA treatment involves open aneurysm surgery or endovascular aneurysm repair [4]. Segmentation of the AAA is instrumental during such surgical interventions, enabling accurate measurement of the precise location and size of the aneurysm [5].

Segmentation techniques can be categorized into manual, semiautomatic, and automatic. Manual segmentation requires the user to delineate the region of interest by setting start-end points or detecting edge signals, whereas semiautomatic segmentation allows the computer to automatically expand the region from a user-defined seed point [6]. However, manual segmentation is time-consuming and suffers from poor reproducibility, while semiautomatic methods require user intervention and can produce variable results depending on the placement of the seed point [7]. Furthermore, accurate segmentation of AAAs is challenging due to their considerable variability in size, curvature, and shape [8]. To address these issues, an automatic segmentation approach based on deep learning has been proposed [9].

U-Net has demonstrated superior segmentation performance by extracting detailed image features through data augmentation [10]. U-Net is composed of contracting and expanding paths. In the contracting path, the features of the image are extracted using multiple down sampling blocks, whereas in the expanding path, the spatial resolution of the feature maps is increased through upsampling blocks [11]. When performing segmentation with U-Net, the presence of noise in the input images can affect segmentation performance [12]. Therefore, removing noise from images before proceeding with segmentation is crucial. Filtering is commonly used to eliminate noise from images, and is typically categorized into local filters such as average, median, and Wiener, and hybrid approaches like the median-modified Wiener filter (MMWF) [13,14,15,16]. This approach has been shown in prior literature to be effective particularly against salt-and-pepper noise due to its edge-preserving properties [16,17]. In this study, we do not propose a novel filter design, but rather evaluate the effect of applying the MMWF as a preprocessing step for improving segmentation performance in noisy abdominal CT images—a combination that has been underexplored in previous studies. While convolutional networks such as U-Net inherently perform both linear and nonlinear operations, we applied traditional filtering methods as a preprocessing step to isolate and analyze the effect of external noise removal independently from the network’s learning process. This approach allows us to evaluate the pure impact of preprocessing filters such as the MMWF on segmentation performance under controlled noise conditions.

Typically, the normal abdominal aortic diameter ranges from 20 to 25 mm, and an aneurysm is diagnosed when the diameter exceeds 30 mm [18]. The risk of rupture rises as the diameter of the aorta increases, and rupture can lead to intraperitoneal hemorrhage with a mortality rate of approximately 65–85% [19]. Surgical intervention is generally indicated for AAAs exceeding 55 mm in diameter [20]. Therefore, accurate measurement of the AAA diameter is crucial. Furthermore, Claridge et al. revealed that radiologists could successfully diagnose only 65% of AAAs, highlighting the need for algorithms that can automatically measure AAA diameter and classify their severity [21]. The Hough circle algorithm, which uses the duality between points on a curve and its parameters to detect curves, can identify circles and measure their diameters [22]. Thus, applying the Hough circle algorithm to automatically detect AAAs and measure their diameters is expected to enable severity classification based on their diameter.

Therefore, this study applied various types of filters to the input images, followed by segmentation based on the U-Net architecture, to quantitatively evaluate the degree of improvement in the segmentation performance attributable to the filters. Subsequently, the Hough circle algorithm was applied to the segmented images of the AAAs to automatically measure their diameters and classify the severity of the aneurysms based on these measurements. The accuracy of the classification was compared and evaluated.

## 2. Materials and Methods

### 2.1. Compliance with Ethical Standards

This study was conducted in accordance with the principles of the Declaration of Helsinki and was approved by the Hallym hospital Institutional Review Board (IRB No. 2025-2).

### 2.2. Acquisition and Filtering of AAA Images

Imaging was performed in 35 patients with AAA using an Aquilion computed tomography (CT) device (Canon Medical Systems, Tustin, CA, USA) with a tube current of 80 mA, tube voltage of 120 kV, and slice thickness of 3 mm. All images used in this study were non-contrast CT scans acquired without the use of intravenous contrast agents. The original CT images were acquired at a resolution of 512 × 512 pixels. For preprocessing, all axial slices were uniformly resized to 256 × 256 pixels using bicubic interpolation in MATLAB R2023a. This resizing was performed to optimize training efficiency while minimizing the loss of structural information, with the understanding that spatial resolution may be slightly degraded. The selected resolution reflects a balance between model performance, computational cost, and GPU memory constraints. This downscaling process was non-destructive in the context of model training and was applied solely for research purposes, not for clinical diagnosis. Following acquisition, Poisson–Gaussian noise was artificially added to the images to amplify the effect of noise. Specifically, Gaussian noise with a standard deviation of 0.05 was added relative to a normalized image intensity range of [0, 1], and the Poisson noise component was applied accordingly. Generally, X-ray images are known to exhibit signal-dependent Poisson noise, while additional noise arises from the detector electronics and other measurement processes [23]. As a result, the observed noise distribution can be reasonably modeled using a Poisson–Gaussian mixture. A general form the signal-dependent noise model is as follows:

(1) I=R+η(R)δ

where I and R represent the acquired and original images, η(R) is the standard deviation of the noise distribution, and δ is the independent Gaussian noise with zero-mean and a standard deviation equal to one. Because the noise depends on the signal, the standard deviation η(R) is defined as a function of the input image R. In this formulation, the total noise variance $\eta^{2}(R)$ can be separated into a signal-dependent term (Poisson) and a signal-independent term (Gaussian). The noise variance is thus described as:

(2) $$\eta^{2}\left(R\right)=\gamma R+\rho^{2}$$

where γ characterizes the contribution of Poisson noise and ρ is the standard deviation of the Gaussian component. The signal-dependent term varies proportionally with the image intensity, while the Gaussian term accounts for constant additive noise from the acquisition system.

This process yielded a final signal-to-noise ratio (SNR) of approximately 13 dB, which represents a decrease from the original image’s estimated SNR of 24 dB based on the acquisition settings. The SNR was calculated by measuring the ratio of mean intensity to standard deviation within a region of interest placed in homogeneous soft tissue regions of the aorta.

After adding the noise, the images were then filtered using four approaches—average, median, Wiener filters, and MMWF—thereby acquiring filtered images. All filtering parameters, including kernel size and noise variance estimates, were applied uniformly across all slices and patients to ensure consistent preprocessing and fair comparison among methods. The average filter is a linear filter that smooths the image by reducing high-frequency components, but often blurs the edges [24]. The median filter, which is a nonlinear method, is effective for removing salt-and-pepper noise while preserving the edges [25]. The Wiener filter used in this study is the standard implementation that estimates local mean and variance within a sliding window, and applies filtering based on these statistics. We note that this differs from the adaptive Wiener filter, which dynamically adjusts parameters based on local signal-to-noise characteristics. Our implementation follows the classical Wiener approach [26]. The MMWF combines the edge-preserving property of the median filter with the adaptive noise-reduction capability of the Wiener filter, offering a balanced denoising approach that preserves structural details while effectively suppressing noise [27].

(3) $$I_{average\left(x,y\right)}=\sum_{i}\sum_{j}I\left(x-i,y-i\right)\;K\left(i,j\right)\;\;$$

(4) $$I_{median\left(x,y\right)}=med\left[I\left(x-i,y-j\right)\;|\left(i,j\right)\;\in \;W\right]$$

(5) $$I_{Wiener\left(x,y\right)}=\mu_{L}+\frac{\sigma_{L}^{2}-\sigma_{n}^{2}}{\sigma_{L}^{2}}\left(I-\mu_{L}\right)$$

(6) $$I_{MMWF\left(x,y\right)}=m_{L}+\frac{\sigma_{L}^{2}-\sigma_{n}^{2}}{\sigma_{L}^{2}}\left(I-m_{L}\right)$$

where $I\left(i,j\right)$ is the input image intensity at location (x,y) and W is the set of pixel indices in the local filtering window. $K\left(i,j\right)$ is the weighting kernel used in the average filter. $\mu_{L}$ and $m_{L}$ denote the local mean and local median within the window W. $\sigma_{L}^{2}$ is the local intensity variance computed within W and $\sigma_{n}^{2}$ is the estimated noise variance.

### 2.3. U-Net-Based Segmentation Model Modeling

**Figure 1:**
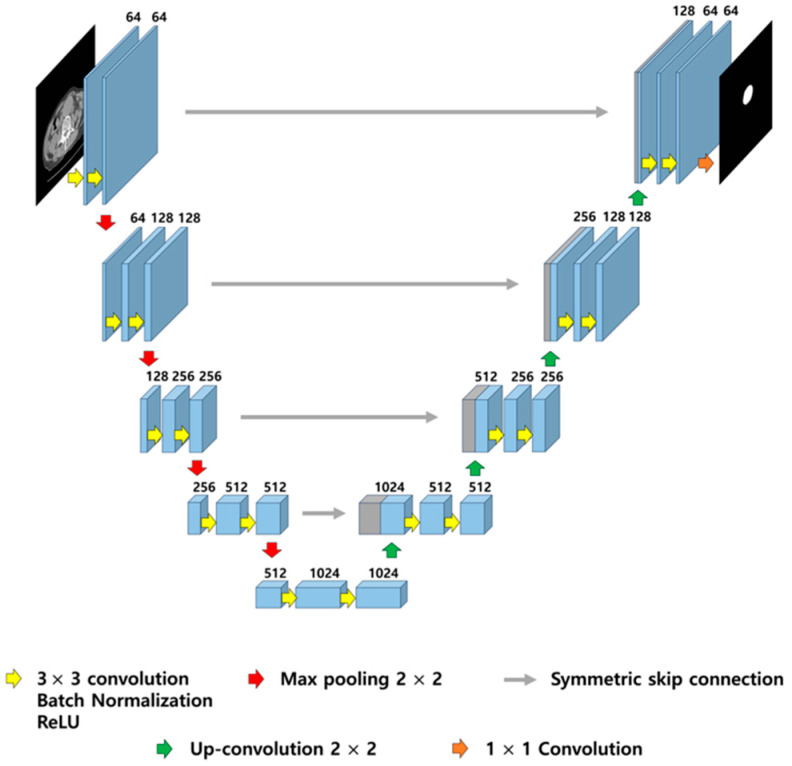
Illustration of U-Net architecture. The model consists of a contracting path (**left**) for feature extraction and an expansive path (**right**) for precise localization, connected by symmetric skip connections to preserve spatial resolution. Each block includes 3 × 3 convolutions with batch normalization and ReLU activation, followed by 2 × 2 max pooling or up-convolution.

In this study, the U-Net architecture was kept fixed to avoid introducing variables from architectural changes or kernel adaptations. Instead of modifying internal filter kernels for denoising, we focused on external preprocessing using classical filters to directly assess their effectiveness in noise suppression prior to training and inference. U-Net training was performed using an NVIDIA GeForce RTX 2080 Ti GPU (NVIDIA Corporation, Santa Clara, CA, USA), and Table 1 lists the parameters used for the training. The U-Net model was implemented in PyTorch 2.2.0 and trained using the Adam optimizer (learning rate = 0.001) and mean squared error loss function for 200 epochs, without applying additional data augmentation. This choice of loss function enables the model to output continuous-valued probability maps, reflecting voxel-wise confidence for aneurysm segmentation, instead of binary masks. Figure 1 illustrates the architecture of the U-Net. The dataset comprised 3062 pairs for training, 802 pairs for validation, and 186 pairs for testing. To prevent data leakage and ensure proper generalization, the dataset was split using a patient-wise separation strategy, where all images from a given patient were assigned exclusively to one of the three subsets (training, validation, or testing). The total dataset comprised 4050 image-mask pairs obtained from 35 patients diagnosed with abdominal aortic aneurysm. Each patient contributed between 80 and 140 axial CT slices, depending on the scan range and aneurysm extent. On average, each patient provided approximately 116 slices. The resulting diversity in anatomical presentation and noise distribution contributed to the robustness of the segmentation model. Images with added noise, as well as those processed with the average, median, Wiener, and MMWF methods, were used as input images for training, whereby the output images were derived for each case. The training progress was monitored using the mean squared error loss and peak signal-to-noise ratio (PSNR) on both training and validation datasets across epochs. The resulting performance curves, along with the test loss and PSNR trends, are provided in Supplementary Figure S1 to demonstrate the stability and convergence of the model throughout training and evaluation.

### 2.4. Quantitative Evaluation of Segmentation Performance

To quantitatively assess the accuracy of the segmentation in the output images derived from the U-Net, the Matthews correlation coefficient (MCC), Dice score (DSC), Jaccard coefficient (JC), and mean surface distance (MSD) were measured using the formulas presented in Equations (7)–(10) [28,29]. The MCC was used to measure the correlation between the segmented pixels and actual values. The DSC represents the harmonic mean of precision and sensitivity and serves as an indicator of the spatial overlap between the segmentation results and actual values. JC indicates the similarity between the segmentation results and actual values. MSD, as an indicator of the average surface distance between the segmentation results and actual values, implies that lower values denote a closer proximity of the segmentation results to the actual values.

(7) $$\;MCC=\frac{TP\times TN-FP\times FN}{\sqrt{\left(TP+FP\right)\left(TP+FN\right)\left(TN+FP\right)\left(TN+FN\right)}}$$

(8) $$DSC=\frac{2\times TP}{2\times TP+FP+FN}$$

(9) $$JC=\frac{DSC}{2-DSC}$$

(10) $$MSD=\frac{1}{N}\sum_{i=1}^{N}d_{i}$$

where TP represents true positives, TN denotes true negatives, FP denotes false positives, and FN denotes false negatives. N is the total number of pixels in the segmented output image, and $d_{i}$ represents the distance from a pixel in the segmented output image to the nearest pixel in the corresponding ground-truth mask image.

Statistical significance of performance differences among filters was assessed using one-way ANOVA followed by post hoc comparisons (Tukey HSD), with significance set at *p* < 0.05.

### 2.5. Classification of AAA Severity Using the Hough Circle Algorithm

The Hough circle algorithm was used to detect circles within an image by employing the Hough circle transform. The principle of the Hough circle transform involves initially detecting the edges in the input image and supporting the possible circle center coordinates (x, y) and radii for each edge pixel. The circle with the highest support in terms of center coordinates and radius was identified, thereby detecting circles within the image [30]. In this study, the Hough circle algorithm was applied to the images segmented by each filter to automatically detect abdominal aortic aneurysms and measure their diameters. Despite its geometric limitations, the Hough circle transform was chosen for its simplicity and alignment with diameter-based clinical classification. It served as a consistent and reproducible method to assess how segmentation quality affects downstream severity classification.

In this study, the severity of the aneurysm was classified into three categories based on the absolute aortic diameter: low risk (<30 mm), moderate risk (30–55 mm), and high risk (>55 mm). These thresholds were selected based on clinical practice guidelines and surgical intervention criteria outlined in prior studies [20,21]. Although BSA-based normalization has been proposed to better account for inter-individual variability, especially in female patients and small body habitus, we used absolute diameter thresholds due to the homogeneity of our dataset and to maintain comparability with previous literature.

## 3. Results

### 3.1. Segmentation Results Using U-Net After Filtering

**Figure 2:**
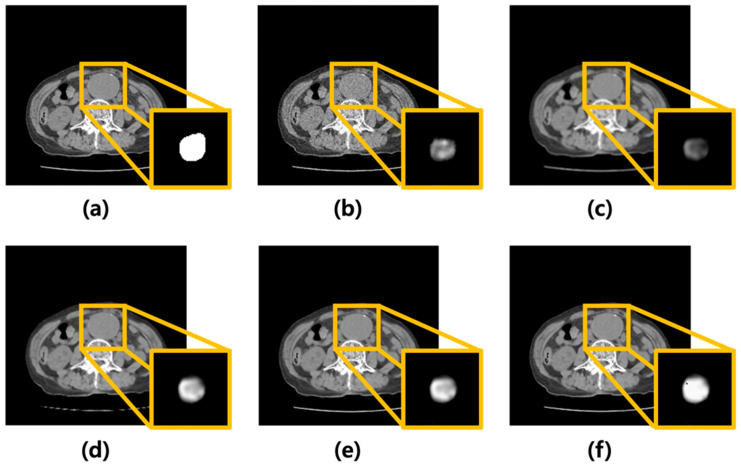
Visual comparison of denoising performance and corresponding segmentation results using different filtering techniques. (**a**) Original abdominal aortic aneurysm CT image. (**b**) Image with added Poisson–Gaussian noise. Images (**c**–**f**) represent the results after applying different denoising filters to the noisy input: (**c**) average, (**d**) median, (**e**) Wiener, and (**f**) median-modified Wiener filters. For each case, the yellow box highlights the region of the abdominal aortic aneurysm. The bottom-right inset in each panel displays the U-Net output for the corresponding denoised image, visualized as a continuous-valued probability map ranging from 0 to 1. These maps reflect voxel-wise confidence of aneurysm segmentation and are visualized in grayscale for consistency across cases.

Poisson–Gaussian noise was added to CT images of AAA using MATLAB. Subsequently, the average, median, Wiener filters, and MMWF were modeled and applied. Figure 2 shows the corresponding images. Figure 2a presents the original CT image of an AAA, while Figure 2b shows the image after noise addition. Figure 2c–f show the results after applying the average, median, Wiener, and MMWF filters, respectively. The bottom-right inset in each filtered image represents the corresponding U-Net segmentation output.

To assess the reliability of the manual annotations used as ground truth, inter-rater agreement was evaluated using the Intra-Class Correlation Coefficient (ICC). Three radiologic technologists independently annotated abdominal aortic aneurysms in CT images. The ICC(2,1) value calculated for the segmentation masks was 0.902 (95% CI: 0.852–0.940), indicating excellent agreement among annotators. These results support the consistency and reliability of the expert-generated ground truth labels used for training and evaluation.

**Figure 3:**
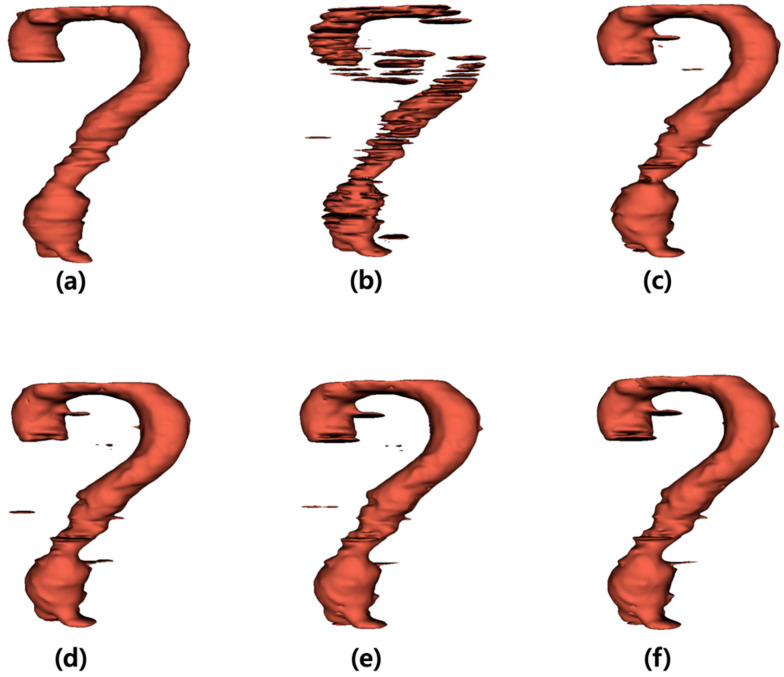
Three-dimensional (3D) visualization of segmentation results for abdominal aortic aneurysm after applying different denoising filters. (**a**) Ground truth 3D model reconstructed from manually segmented label data. (**b**) 3D model generated from noisy input image, (**c**–**f**) 3D models obtained from U-Net outputs after applying (**c**) average, (**d**) median, (**e**) Wiener, and (**f**) median-modified Wiener filter, respectively.

The output images generated by the U-Net were summed along the *z*-axis to construct three-dimensional (3D) models (Figure 3). Figure 3a illustrates the 3D model of the label data, and Figure 3b presents the 3D reconstruction of the output derived from the noisy input image. Figure 3c–f shows the 3D models of the output images obtained from the inputs processed with the average, median, Wiener, and MMWF filters, respectively.

**Figure 4:**
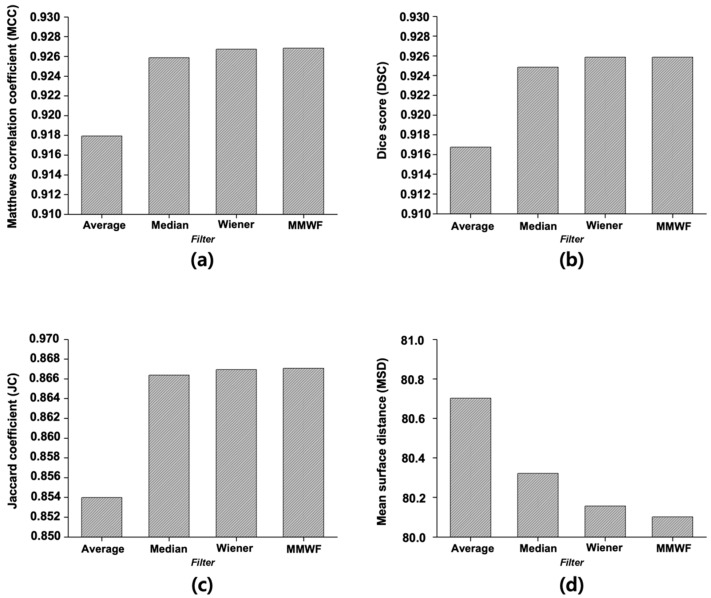
Quantitative evaluation of segmentation results using different denoising filters based on four performance metrics: (**a**) Matthews correlation coefficient, (**b**) Dice score, (**c**) Jaccard coefficient, and (**d**) Mean surface distance. Each metric was calculated to assess the accuracy and structural consistency of the U-Net segmentation outputs. Filters compared include average, median, Wiener, and median-modified Wiener filters.

Figure 4 displays the quantitative evaluation results of segmentation performance, calculated as the average scores across 50 representative CT slices. For the image with added noise, the MCC was 0.7070. When the average, median, Wiener, and MMWF filters were applied, the MCC increased to 0.9179, 0.9259, 0.9267, and 0.9268, respectively. The DSC for the noisy image was 0.6897, whereas the values for the average, median, Wiener, and MMWF filters were 0.9168, 0.9249, 0.9258, and 0.9259, respectively. The JC value was 0.5631 for the noisy input and improved to 0.8540, 0.8664, 0.8669, and 0.8671 for the average, median, Wiener, and MMWF filters, respectively. Finally, the MSD was 83.06 for the noisy image and decreased to 80.70, 80.32, 80.16, and 80.10 after applying the average, median, Wiener, and MMWF filters, respectively. One-way ANOVA revealed statistically significant differences in MCC, DSC, JC, and MSD scores across the four filtering methods (*p* < 0.001 for all). Post hoc comparisons indicated that the MMWF significantly outperformed the other filters (Supplementary Table S1).

### 3.2. Severity Classification of AAAs Using the Hough Circle Algorithm

Following segmentation, the diameter of the aneurysm was measured using the Hough circle algorithm on the output images obtained from each filter, and severity was classified based on the measured diameter. Table 2 summarize the classification results. The sensitivity, precision, and accuracy of the images with added noise were 89.67%, 78.47%, and 89.67%, respectively. When the average filter was applied, these values were 94.74%, 88.57%, and 89.67%. For the images processed using the median filter, the corresponding values were 96.49%, 95.24%, and 96.49%, whereas the Wiener filter yielded 98.25%, 97.43%, and 98.25%, respectively. The application of the MMWF filter resulted in 100% sensitivity, 100% precision, and 100% accuracy.

## 4. Discussions

The risk factors for AAA include smoking, age > 65 years, hypertension, coronary artery disease, and family history. Among individuals aged 74–84 years, 12.5% of men and 5.2% of women are affected by AAA [31]. In the United States, AAA accounts for approximately 11,000 deaths annually [32]. Since most AAAs are asymptomatic, increasing the diagnostic rate through screening is important [33]. CT is widely used to diagnose AAAs because the modality provides clear visualization. Currently, CT is mostly used for preoperative evaluation because it enables stable visualization of the lumen and thrombus, allowing accurate measurement of the aneurysm size. Through CT imaging of AAAs, the size of the aneurysm, presence of anatomical deformities, and the presence of inflammation or fibrosis around the aneurysm can be identified, all of which are important for surgical planning [34].

AAA severity can be classified based on size, and the risk of rupture increases with larger diameters [33]. When the diameter exceeds 55 mm, treatment is usually performed via open surgery or endovascular aortic repair (EVAR) [35]. Postoperative monitoring helps assess surgical success and detect recurrence. Accurate AAA segmentation is crucial during surgical procedures. Segmentation is necessary to precisely determine the size, shape, location, and branching points of the aneurysm preoperatively and is important for evaluating postoperative changes in aneurysm size and morphology. Postoperative monitoring helps assess surgical success and detect recurrence.

Various deep learning-based segmentation methods have been proposed to segment AAAs. Recently, deep learning-based segmentation has been widely applied in the medical imaging field and is effectively used for diagnosis and quantitative organ analysis. However, significant limitations in achieving accurate segmentation persist due to low contrast, intensity inhomogeneity, and noise. In particular, CT images inevitably contain noise generated during image acquisition, such as amplifiers, shots, grains, and electrical interference [36]. Furthermore, deep learning-based segmentation requires large-scale training datasets, and ideal reference images are essential for model training [37]. However, in clinical practice, acquiring CT images inevitably introduces noise, limiting the acquisition of ideal reference images and difficult-to-obtain large-scale datasets. Furthermore, the black-box characteristics of deep learning lead to ambiguous result interpretation, which can undermine diagnostic reliability in clinical settings [38]. Therefore, this study selected the MMWF algorithm, which is based on mathematical algorithms, ensures transparent processing with guaranteed reproducibility and consistency, and can be applied using single data. In our study, we achieved effective noise reduction and successful AAA segmentation. Based on our experimental results, if the MMWF algorithm can be applied to data preprocessing for deep learning model training, it is expected to improve the dataset quality for deep learning models, thereby further enhancing learning stability and segmentation accuracy. Moreover, we believe the MMWF algorithm can be effectively utilized as a data preprocessing step for AAA images as well as for segmenting other organs and lesions. The preprocessing steps for CT images include Hounsfield unit (HU) windowing, histogram equalization, and filtering [39]. Adjusting the HU values is essential to clearly visualize the target organ. Moreover, when the image contrast is low, the image quality may be degraded, and important information is obscured. Therefore, histogram equalization must be applied to enhance contrast [12]. If noise is present in an image, the noise interferes with deep learning-based segmentation and degrades the overall image quality [39]. Thus, noise removal through filtering is important, as it improves the signal-to-noise ratio and contrast-to-noise ratio, thereby enhancing segmentation accuracy.

**Figure 5:**
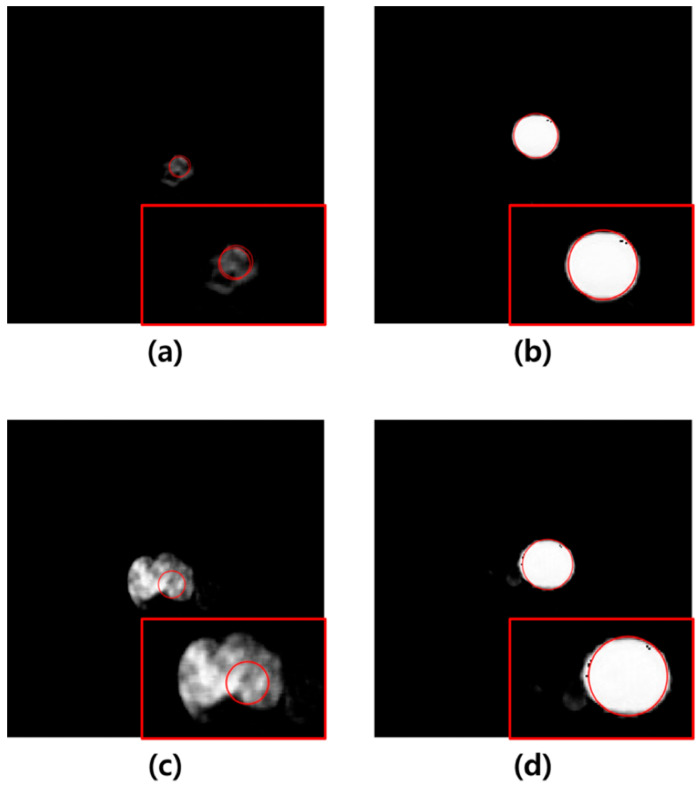
(**a**) Example of a medium-risk case misclassified as low-risk in a noisy image. (**b**) Example of a medium-risk case correctly classified in an image preprocessed with the MMWF filter. (**c**) Example of a high-risk case misclassified as medium-risk in a noisy image. (**d**) Example of a high-risk case correctly classified in an image preprocessed with the MMWF filter. In each image, the red box shows a magnified view of the segmented abdominal aortic aneurysm, and the red circle indicates the boundary detected by the Hough circle algorithm, which is used for risk classification based on diameter.

In this study, U-Net-based segmentation was performed using images with added noise and those filtered using the average, median, Wiener, and MMWF as inputs. Although the convolutional layers of U-Net can inherently perform noise suppression via learned filters, this effect can vary significantly depending on training conditions, data volume, and noise type. Therefore, we applied external filtering methods to provide consistent and interpretable comparisons across different denoising techniques without altering the core model architecture. The MCC, DSC, JC, and MSD were used to evaluate the segmentation accuracy of the output images. The results showed that the highest segmentation performance was achieved when the MMWF-filtered images were used as the input. Specifically, compared to noisy input images, the MMWF-filtered images improved the MCC, DSC, JC, and MSD by 31.09%, 34.25%, 53.99%, and 3.70%, respectively. While the MMWF itself is not a novel algorithm, its application to enhance segmentation of abdominal CT images in the presence of synthetic Poisson–Gaussian noise has not been systematically evaluated in previous studies. This work contributes to the field by demonstrating that classical denoising filters can substantially improve the performance of deep learning-based segmentation pipelines when used appropriately during preprocessing. Using the segmented images, the Hough circle algorithm was applied to measure the diameter, and severity was classified based on the diameter. The classification results demonstrated that the highest accuracy was achieved when using the output images derived from the MMWF-filtered inputs. Compared to classification using output images from noisy inputs, the sensitivity, precision, and accuracy improved by 10.33%, 21.53%, and 10.33%, respectively. This confirms that higher segmentation accuracy leads to more accurate severity classification. Although the aortic lumen may appear clearly delineated in certain axial slices, particularly in the mid-abdominal region, our dataset included numerous challenging cases that complicated segmentation. These included slices with irregularly shaped aneurysms, overlapping soft tissues, adjacent organ artifacts, and reduced contrast between the vessel wall and surrounding structures—especially in the absence of intravenous contrast. In such cases, even learning-based methods such as U-Net showed suboptimal performance when trained and tested on noisy input images. Figure 5 illustrates failure cases where segmentation errors due to noise directly led to incorrect severity classification. These examples underscore the importance of applying effective denoising filters prior to segmentation, as such preprocessing can significantly improve robustness in anatomically and visually complex scenarios. In Figure 5a, severity classification was performed using the output image derived from the noisy input, and a moderate-risk AAA was misclassified as low-risk due to reduced segmentation accuracy. Conversely, Figure 5b shows that the output image obtained from the MMWF-filtered input led to the correct classification of moderate-risk. Similarly, in Figure 5c, the output image from the noisy input led to the misclassification of a high-risk AAA as moderate-risk, whereas in Figure 5d, the MMWF-filtered input resulted in the correct classification as high-risk.

However, this study had several limitations. First, the CT images used for evaluation were artificially corrupted with Poisson–Gaussian noise to simulate realistic conditions. While this noise model approximates some characteristics of clinical degradation, it does not fully capture the spatially correlated and nonstationary nature of noise introduced during the projection and reconstruction processes in actual CT imaging. Therefore, further validation using real low-dose or noisy CT data is essential to confirm the generalizability of our findings. Second, the dataset used for training and evaluation was relatively small and derived from a limited number of subjects. Consequently, the generalizability of the segmentation model may be restricted when applied to external datasets or images acquired with different CT scanners or protocols. Furthermore, although the MMWF filtered images achieved near perfect classification metrics in this study, these results should be interpreted with caution. The small sample size consisting of 35 patients, internal-only validation, and potential label bias due to annotations created on inherently noisy images may contribute to overly optimistic performance estimates. Third, this study did not include comparisons with more advanced deep learning based denoising or end-to-end noise-robust segmentation models. Future work should explore these methods and benchmark them against our MMWF based preprocessing to better contextualize its utility. In addition, investigating how varying noise levels simulating dose reduction affect segmentation accuracy may help answer an important clinical question: how much can CT dose be lowered without compromising AI based performance? Fourth, the Hough circle algorithm used for AAA classification is inherently limited by its assumption of a circular geometry. AAAs can be broadly classified into the mycotic and atherosclerotic types. Mycotic aneurysms typically occur in individuals with intravenous drug use, alcohol abuse, or immunocompromised conditions, and may arise in patients with congenital or post-traumatic vascular abnormalities [40]. These aneurysms have a high risk of rupture, are associated with high mortality rates, and require immediate surgical intervention. However, due to their irregular shapes, accurate diameter measurements using the Hough circle algorithm are often difficult [1]. While atherosclerotic aneurysms generally exhibit a spherical morphology, they can also present various patterns, limiting the accuracy of diameter measurements based solely on the Hough circle algorithm [41]. As AAA morphology ranges from perfectly spherical to fusiform or saccular, accurate detection becomes challenging when aneurysms deviate from a circular form. Future research should address these limitations through multiple directions. Expanding the dataset to include multicenter clinical images with diverse acquisition parameters improved the robustness and clinical applicability of the model. In our analysis, 7 out of 35 cases resulted in incorrect severity classification when the Hough circle algorithm was applied to segmentation masks derived from noisy or poorly filtered images. These errors primarily occurred in slices with irregularly shaped or elongated aneurysms, where the circularity assumption failed. To address such limitations, future work should explore more adaptive geometric models such as ellipse fitting, active contour methods, or deep shape encoders that can better capture the anatomical variability of AAAs. Moreover, an end-to-end deep learning severity classifier trained directly on segmentation outputs or raw images may offer a more holistic and robust alternative to diameter-based rule-based classification. Additionally, our classification method used fixed diameter thresholds without normalization to body surface area (BSA). While such thresholds are consistent with clinical guidelines for surgical decision-making, BSA-normalized aortic diameter may offer more individualized risk assessment. Future studies should consider incorporating BSA-adjusted diameter metrics to improve generalizability and applicability in more diverse patient populations. Although our study does not propose a novel network architecture or denoising model, it contributes by systematically evaluating how classical filtering methods, particularly the MMWF, impact segmentation and classification performance under noise-degraded conditions. This application-driven framework highlights the practical utility of lightweight preprocessing strategies in enhancing robustness without increasing model complexity or retraining requirements. Finally, integrating the proposed segmentation and classification framework into a fully automated, real-time clinical decision support system may enable the early detection, treatment planning, and monitoring of AAAs in various healthcare settings.

## 5. Conclusions

In this study, we applied various types of filters to the input images before performing U-Net-based segmentation to quantitatively evaluate the extent of segmentation performance improvement according to each filter. Subsequently, we assessed the accuracy of severity classification based on the segmentation results using the Hough circle algorithm. The results confirmed that both segmentation performance and classification accuracy generally improved when filtered images were used as inputs compared with noisy images. Among the filters, the MMWF yielded the most significant improvements in both segmentation accuracy and severity classification. Therefore, this study demonstrated that applying the MMWF during the preprocessing stage, followed by U-Net-based segmentation and the Hough circle algorithm, can enhance AAA classification accuracy.

## Figures and Tables

**Table 1:** Hyperparameters and configuration details of the U-Net architecture.

Parameter	Dimension
Size of input and output images	256 × 256
Training data	3062
Validating data	802
Testing data	186
Number of epochs	200
Size of batch	3
Number of channels	64, 128, 256, 512, and 1024
Learning rate	5 × 10^−4^
Objective function	Mean Squared Error Loss
Optimization solver	Adaptive momentum estimation (Adam)

**Table 2:** Severity classification results for images segmented with noisy input.

Filter	Unit: mm	D < 30	30 ≤ D ≤ 55	D > 55	Sensitivity (%)	Precision (%)	Accuracy (%)
Noisy	D < 30	4	1	1	100	66.67	100
	30 ≤ D ≤ 55	0	11	5	91.67	68.75	91.67
	D > 55	0	0	13	68.42	100	68.42
	Total	4	12	19	89.67	78.47	89.67
Average	D < 30	4	1	1	100	66.67	100
	30 ≤ D ≤ 55	0	11	5	91.67	68.75	91.67
	D > 55	0	0	13	68.42	100	68.42
	Total	4	12	19	89.67	78.47	89.67
Median	D < 30	4	0	1	100	100	100
	30 ≤ D ≤ 55	0	12	2	100	85.71	100
	D > 55	0	0	17	89.47	100	89.47
	Total	4	12	19	96.49	95.24	96.49
Wiener	D < 30	4	0	0	100	100	100
	30 ≤ D ≤ 55	0	12	1	100	92.3	100
	D > 55	0	0	18	94.74	100	94.74
	Total	4	12	19	98.25	97.43	98.25
MMWF	D < 30	4	0	0	100	100	100
	30 ≤ D ≤ 55	0	12	0	100	100	100
	D > 55	0	0	19	100	100	100
	Total	4	12	19	100	100	100

## Data Availability

The data used in this study were obtained from Hallym Hospital and are not publicly available because of institutional privacy regulations. Access to the data may be granted upon reasonable request and with permission from Hallym Hospital’s Institutional Review Board.

## Supplementary Materials

The following supporting information can be downloaded at: https://www.mdpi.com/article/10.3390/s25216509/s1, Figure S1: Training-validation and testing loss/PSNR curves of the U-Net model across epochs; Table S1: ANOVA and post hoc comparison results for segmentation metrics.

## Abbreviations

The following abbreviations are used in this manuscript:

AAA	Abdominal aortic aneurysm
MMWF	Median-modified Wiener filter
CT	Computed tomography
MCC	Matthews correlation coefficient
DSC	Dice score
JC	Jaccard coefficient
MSD	Mean surface distance
ICC	Intra-class correlation coefficient
3D	Three-dimensional
EVAR	Endovascular aortic repair
BSA	Body surface area
