# Supplementary material for: Modeling of Severity Classification Algorithm Using Abdominal Aortic Aneurysm Computed Tomography Image Segmentation Based on U-Net with Improved Noise Reduction Performance
Source: Sensors (Basel). 2025 Oct 22;25(21):6509. doi: 10.3390/s25216509 (PMC12610145; doi:10.3390/s25216509)

Supplemental Materials

Supplemental Table S1. ANOVA and post hoc comparison results for segmentation metrics.

Supplemental Figure S1. Training–validation and testing loss/PSNR curves of the U-Net model across epochs.

Supplemental Table S1. ANOVA and post hoc comparison results for segmentation metrics.

Metric	F-value	P-value	Significant Post hoc comparisons (P < 0.05)
**MCC**	869.4	< 0.001	MMWF > Average, Median, Wiener
**DSC**	2215.8	< 0.001	MMWF > Average, Median, Wiener
**JC**	1642.9	< 0.001	MMWF > Average, Median, Wiener
**MSD**	34.9	< 0.001	MMWF < Average, Median, Wiener

MCC, Matthews correlation coefficient; DSC, Dice score; JC, Jaccard coefficient; MSD, Mean surface distance.

Supplemental Figure S1. Training–validation and testing loss/PSNR curves of the U-Net model across epochs : (a) Training and validation loss, (b) Training and validation PSNR, (c) Testing loss, and (d) Testing PSNR.

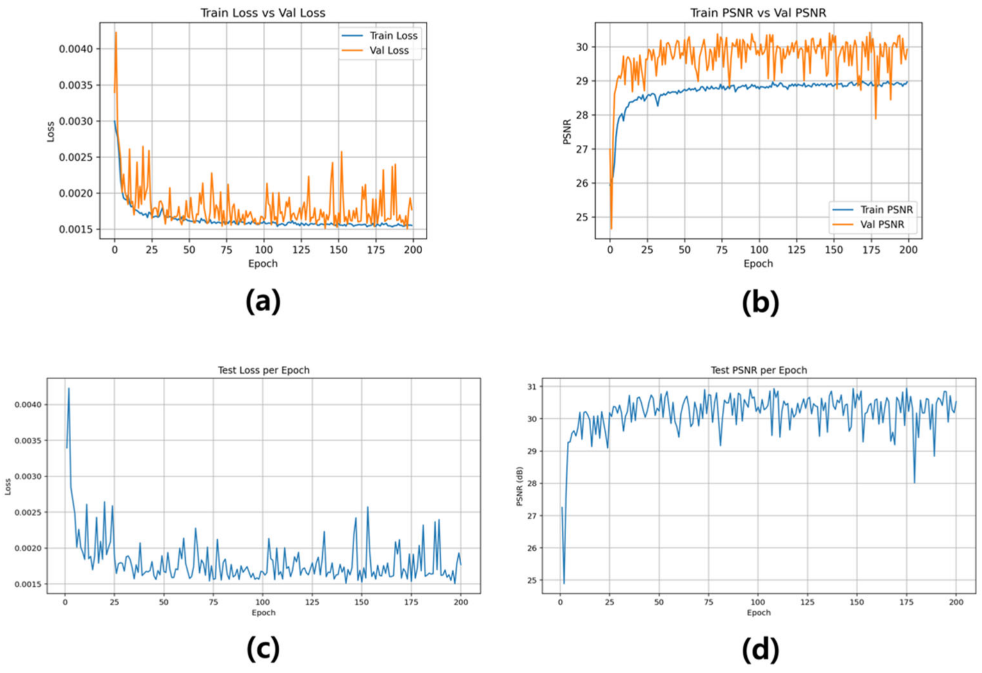

Supplement: Supplementary file 1 [file sensors-25-06509-s001.zip › sensors-3903104-supplementary.pdf]
